# Double-Edge Effects of Leucine on Cancer Cells

**DOI:** 10.3390/biom14111401

**Published:** 2024-11-04

**Authors:** Burkitkan Akbay, Zhannur Omarova, Alexander Trofimov, Bayan Sailike, Orynbassar Karapina, Ferdinand Molnár, Tursonjan Tokay

**Affiliations:** Department of Biology, School of Sciences and Humanities, Nazarbayev University, Kabanbay Batyr 53, Astana 010000, Kazakhstan; burkitkan.akbay@nu.edu.kz (B.A.); zhannur.omarova@nu.edu.kz (Z.O.); aleksandr.trofimov@nu.edu.kz (A.T.); bayan.sailike@alumni.nu.edu.kz (B.S.); orynbassar.karapina@nu.edu.kz (O.K.); ferdinand.molnar@nu.edu.kz (F.M.)

**Keywords:** amino acid, leucine, muscle protein, cancer, signaling pathway

## Abstract

Leucine is an essential amino acid that cannot be produced endogenously in the human body and therefore needs to be obtained from dietary sources. Leucine plays a pivotal role in stimulating muscle protein synthesis, along with isoleucine and valine, as the group of branched-chain amino acids, making them one of the most popular dietary supplements for athletes and gym-goers. The individual effects of leucine, however, have not been fully clarified, as most of the studies so far have focused on the grouped effects of branched-chain amino acids. In recent years, leucine and its metabolites have been shown to stimulate muscle protein synthesis mainly via the mammalian target of the rapamycin complex 1 signaling pathway, thereby improving muscle atrophy in cancer cachexia. Interestingly, cancer research suggests that leucine may have either anti-cancer or pro-tumorigenic effects. In the current manuscript, we aim to review leucine’s roles in muscle protein synthesis, tumor suppression, and tumor progression, specifically summarizing the molecular mechanisms of leucine’s action. The role of leucine is controversial in hepatocellular carcinoma, whereas its pro-tumorigenic effects have been demonstrated in breast and pancreatic cancers. In summary, leucine being used as nutritional supplement for athletes needs more attention, as its pro-oncogenic effects may have been identified by recent studies. Anti-cancer or pro-tumorigenic effects of leucine in various cancers should be further investigated to achieve clear conclusions.

## 1. Introduction

Amino acids (AAs) are organic molecules containing an α-carboxyl group, an α-amino group, and a side chain, the R group. Due to the variations in their R group, amino acids present remarkably different biochemical characteristics and functions. Among about 500 amino acids that have been identified in nature, 20 AAs (4% of all amino acids) are known to be involved in assembling proteins, one of the major building blocks of life [1]. Therefore, amino acids play a myriad of roles—functional, structural, metabolic, and beyond—and remain exceptionally important in maintaining overall human health. To name a few, amino acids are known to play a fundamental role in skeletal muscle health in aging, in cardiovascular physiology and pathology, in intestinal physiology and health, in endocrine function, and in immune response regulation [2,3,4,5,6]. These 20 proteinogenic (protein assembling) amino acids are further classified as nonessential (11 amino acids) and essential amino acids (9 amino acids). Nonessential amino acids are synthesized in the body, while the essential ones must be supplemented via diet.

Of the nine essential amino acids—histidine, isoleucine, leucine, lysine, methionine, phenylalanine, threonine, tryptophan, and valine—leucine (Leu), isoleucine (Ile), and valine (Val) are grouped as branched-chain amino acids (BCAAs) due to their aliphatic side chains, which are branched (hence their name), small, and hydrophobic [7]. Because of their similarities in metabolism, consumption, and combustion, BCAAs have always been studied together [8]. Unlike most amino acids, which are catabolized in the liver, the catabolism of all three BCAAs starts in the skeletal muscle and the same enzymes are used for the first two steps [7]. At the first step, branched-chain α-keto acids (BCKAs)—2-ketoisocaproate (KIC) from leucine, 2-keto-3-methylvalerate (KMV) from isoleucine, and 2-ketoisovalerate (KIV) from valine—are generated with the help of branched-chain aminotransferase enzyme, which is a reversible process. The freed amino groups from BCAAs are accepted by 2-ketaglutarate, which yields glutamate, by pyruvate, which yields alanine, and by glutamate, with the addition of another amino group which yields glutamine. The generated BCKAs, glutamate, alanine, and glutamine are further released from the muscle and enter systemic circulation. In the second step, isovaleryl-CoA from KIV (from leucine), 2-methylbutyryl-CoA from KMV (from isoleucine), and isobutyryl-CoA from KIV (from valine) are generated with the help of the branched-chain α-ketoacid dehydrogenase (BCKD). These acyl-CoA metabolites further processed by different enzymes and undergo different breakdown pathways [7,9].

BCAAs have been extensively studied in many diseases, such as liver cirrhosis, renal failure, sepsis, trauma, burn injury, and cancer [10]. Under normal conditions, the levels of BCAAs are maintained in balance by their intake and expenditure. In a fasting state, the average levels of BCAAs in circulation are approximately 200 μM of valine, 100 μM of leucine, and 60 μM of isoleucine [11]. BCAAs obtained from the diet or released from tissues with protein breakdown can be used in protein synthesis. Elevations in circulating BCAAs have been reported in many tumors and BCAAs released from protein degradation or obtained from the tumor microenvironment are also hypothesized to fuel growing cancer cells [11], where BCCAs can serve as nitrogen donors used to produce nucleic acids and other macromolecules that are vital for the proliferation of tumors [12]. BCAAs play a pivotal role in various metabolic reactions and act as biochemical regulators of protein turnover [13,14,15]. BCAAs supplementation has increased in sports nutrition and has become a vital part of the daily diet of bodybuilders and gym-goers, as BCAAs promote protein synthesis, preventing its breakdown induced by intensive exercise [16,17,18]. They also play fundamental role in the muscles’ post-exercise recovery [19,20], and are implicated in the delay of fatigue by reducing the brain’s uptake of tryptophan and synthesis of 5-hydroxytryptamine [21,22]. These functions make BCAAs a “panacea” for professional and amateur athletes. As a result of such attention, the global sales of BCAAs have been steadily increasing from year to year [23,24]. Interestingly, the potential benefits of BCAAs on performance, strength gains, and muscle mass have been a subject of ongoing debate [25,26,27]. Adding to this controversy, recent studies have shown that BCAA metabolism may be also associated with the progression of various types of tumors [28,29].

Even though the three BCAAs exhibit significant differences in their biological effects, they are often studied together as a single group, leading to potentially erroneous assumptions about their individual impacts [8]. Therefore, in this review, we primarily focus on Leu, as it is considered the most influential regulator and signaling molecule among the BCAAs [27,30].

## 2. Leucine and Muscle Protein Synthesis: Mechanisms and Impacts

Leucine was first discovered in cheese in 1819 and was first isolated from skeletal muscle and wool in 1820. More than a century later, leucine was synthesized from isovaleraldehyde [31]. Since then, leucine has been extensively studied in protein synthesis and energy production within cells.

**Figure 1:**
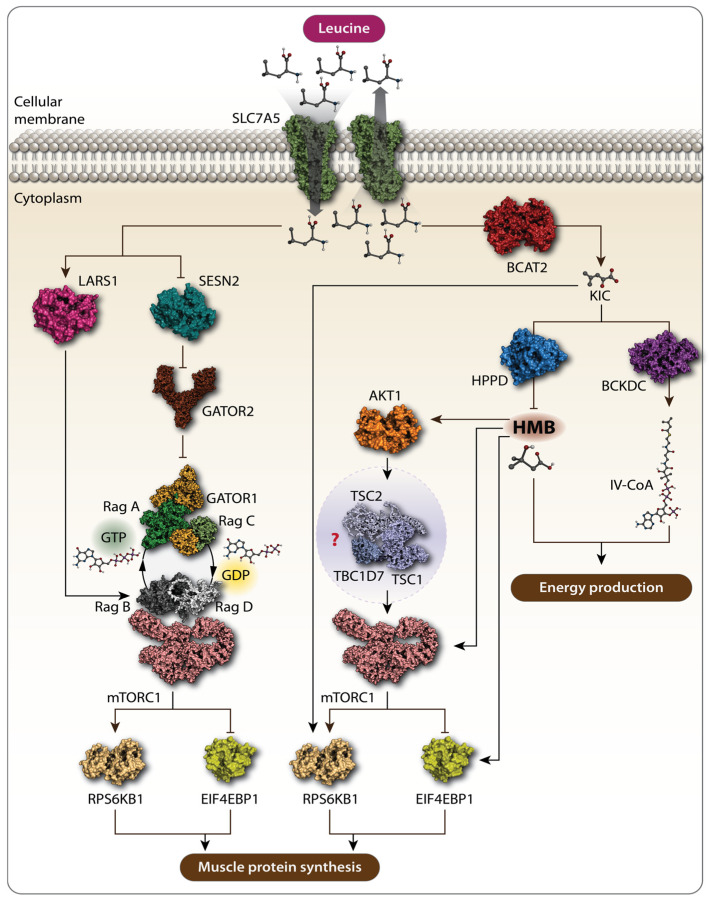
Schematic representation of the action of Leu and its metabolites. Solute carrier family 7 member 5 (SLC7A5) transports extracellular Leu into the cells. Amino acid sensors, namely leucyl-tRNA synthetase (LARS1) and sestrin 2 (SESN2), detect cytosolic Leu. SESN2 alleviates the inhibitory function of GTPase-activating protein toward Rags 1 (GATOR1) and GTPase-activating protein toward Rags 2 (GATOR2), leading to the activation of the mechanistic target of rapamycin complex 1 (mTORC1) signaling pathway, which further promotes muscle protein synthesis (MPS). Leu metabolites also promote MPS via mTORC1. Branched-chain amino transferase 2 (BCAT2) converts Leu to α-ketoisocaproate (KIC), which phosphorylates p70 ribosomal protein S6 kinase 1 (RPS6KB1) and eukaryotic translation initiation factor 4E-binding protein 1 (4E-BP1), both markers of mTORC1 activation, thereby increasing MPS. KIC can be further metabolized to β-hydroxy-β-methylbutyrate (HMB) by KIC dioxygenase and to isovaleryl-CoA by branched-chain α-keto acid dehydrogenase complex (BCKDC). Rag GTPases are crucial in this pathway. RagA/B typically bind GTP when Leu is abundant, while RagC/D are in the GDP-bound state. The RagA–RagC and RagB–RagD heterodimers are key pairs interacting with mTORC1 on the lysosomal membrane, activating it in response to Leu and promoting MPS. Both HMB and isovaleryl-CoA are involved in energy production. HMB increases MPS by activating the AKT1-mTORC1 signaling pathway. However, whether HMB promotes MPS via the AKT1-mTORC1 axis is not fully characterized, as the effect of HMB on the tuberous sclerosis complex 1-tuberous sclerosis complex 2-TBC1 domain family member 7 (TSC1-TSC2-TBC1D7) complex, the intermediate between AKT1 and mTORC1. Arrows stand for “activation”, “stimulation”. Bars stand for “inhibition”. Question mark stands for “uninvestigated”.

The muscle protein mass is maintained by a dynamic equilibrium between muscle protein synthesis (MPS) and muscle protein breakdown (MPB), determining the net protein balance [32]. Leu has been shown to play a particularly central role in MPS [33], stimulating a robust MPS response in humans at a relatively low dose of about 3 g, even in the absence of other amino acids [34]. Unlike other essential amino acids, Leu is mainly metabolized within the skeletal muscle, where, with the help of the mitochondrial branched-chain amino transferase 2 (BCAT2), Leu is converted to its keto-acid, α-ketoisocaproate (KIC), which can be further metabolized either to isovaleryl-CoA by the branched-chain α-keto acid dehydrogenase complex (BCKDC) or to β-hydroxy-β-methylbutyrate (HMB) by KIC dioxygenase, ultimately serving as an energetic substrate for the citric acid cycle to produce energy (Figure 1) [34,35]. Both Leu itself and its metabolites, KIC and HMB, have been shown to increase MPS (Figure 1) [34,36,37]. Due to Leu’s crucial role in regulating protein metabolism, it is used to combat protein loss in patients with various pathological conditions. This is supported by recent trials, which demonstrated that Leu supplementation improved sarcopenia in older adults [38,39,40,41]. The mechanism by which Leu and its metabolites enhance protein synthesis in muscle involves the activation of the mechanistic target of the rapamycin complex 1 (mTORC1) signaling pathway [42,43]. This pathway is a key regulator that integrates various cellular processes, including protein synthesis [44]. mTORC1 senses signals from both intra- and extracellular cues, including the availability of amino acids. As a result, nutrient sensing and responding to their availability are considered the primary functions of mTORC1. Specific transporters are involved in bringing amino acids into the cells. For Leu, the solute carrier family 7 member 5 (SLC7A5)/SLC3A2, a heterodimeric bidirectional transporter, is responsible for transporting extracellular leucine into the cells in exchange for intracellular L-glutamine [45]. Within the cell, the lysosome is considered a key site for amino acid sensing, where specific proteins, known as amino acid sensors, help mTORC1 detect amino acids. For Leu, the major detectors in the cytosol are leucine-tRNA ligase (LARS1) and sestrin 2 (SESN2) [46,47,48,49]. These sensors closely interact with Ras-related guanine triphosphatase (GTP)binding proteins (RagA, B, C, and D), a small GTPase family, as well as GTPase-activating proteins, such as the multiprotein complex GTPase-activating proteins toward Rags (GATOR), such as GATOR1 and GATOR2, in order to communicate information about amino acid availability to mTORC1 [47]. Upon Leu availability, LARS1 specifically interacts with the RagA–RagC or RagB–RagD heterodimers, promoting the proper nucleotide loading state, which is essential for mTORC1 activation. LARS1 functions as a GTPase-activating protein towards RagD–GTP, which further facilitates the activation of the mTORC1 pathway [47]. Additionally, Leu binds to SESN2, causing its dissociation from GATOR2, which removes the inhibitory effect of GATOR1 on mTORC1, ultimately activating the pathway (Figure 1) [50,51]. Once activated, mTORC1 phosphorylates p70 ribosomal protein S6 kinase 1 (RPS6KB1) and eukaryotic translation initiation factor 4E-binding protein 1 (4E-BP1). The phosphorylation of RPS6KB1 enhances its kinase activity, further promoting protein translation, while the phosphorylation of 4E-BP1 prevents its association with eukaryotic translation initiation factor 4E (eIF4E), allowing translation initiation to occur (Figure 1) [52]. Through these mechanisms, Leu increases MPS via the activation of the mTORC1 pathway. However, Leu metabolites appear to be sensed differently from the Leu-sensing pathway described above. For example, KIC has been shown to increase protein synthesis by promoting the phosphorylation of mTORC1 substrates, RPS6KB1 and 4E-BP1 [37,53], although the upstream mechanism by which mTORC1 senses KIC remains unclear. More research has been conducted on HMB, another Leu metabolite. Earlier studies demonstrated that HMB increased the phosphorylation of mTORC1 downstream targets, RPS6KB1 and 4E-BP1, thereby enhancing skeletal MPS [54,55]. An independent study explored how HMB activates nutrient sensing upstream of mTORC1, suggesting that the effects of HMB on MPS may be mediated by the phosphatidylinositol 3-kinase (PI3K)-RAC-α serine/threonine-protein kinase (AKT1)-mTORC1 signaling axis [56]. Although the PI3K-AKT1 signaling pathway primarily transmits signals from growth factors and cytokines to mTORC1, acting as a major upstream regulator of the mTORC1 pathway, this study indicates that HMB might also influence mTORC1 through this pathway (Figure 1) [57,58]. PI3K, when activated in response to growth factors and cytokines, phosphorylates and activates AKT1. Active AKT1 then phosphorylates and inactivates TSC2, a component of the tuberous sclerosis complex (TSC), which includes TSC1, TSC2, and TBC1 domain family member 7 (TBC1D7). The TSC is a negative regulator of mTORC1, so its inactivation leads to the activation of the mTORC1 pathway [59,60]. Girón et al. [56] demonstrated that HMB phosphorylates AKT1, mTOR, and its downstream targets, RPS6KB1 and 4E-BP1, thereby increasing protein synthesis. However, this study did not investigate whether AKT1 activates mTORC1 through the TSC, making it difficult to conclude whether HMB exerts its effects via the PI3K-AKT1-mTORC1 signaling axis. A more recent study provided evidence that HMB stimulates protein synthesis in skeletal muscle by inducing the autophosphorylation of mTOR, leading to the activation of the mTORC1 pathway without involving the SESN2-GATOR2 or Rag GTPase family proteins [43] (Figure 1). These findings suggest that Leu metabolites, such as KIC and HMB, may be sensed independently of the traditional Leu-sensing pathway. However, further research is needed to reach a consensus on the mechanisms by which Leu metabolites influence protein synthesis.

## 3. Leucine and Cancer

As mentioned above, Leu has been shown to increase MPS and MPB [61,62]. In recent years, its potential to improve muscle wasting in cancer cachexia (CC) has been extensively studied [63,64,65]. CC is a complex syndrome characterized by glucose intolerance, loss of body fat, significant body weight loss due to muscle mass depletion, and malnutrition. As a result, nutritional supplementation including Leu has been explored as a novel and promising therapeutic approach to protect from cancer-associated cachexia [66]. Considering that leucine supplementation can improve sarcopenia in the elderly, Herrera-Martínez et al. (2023) recently investigated the feasibility of using a leucine-enriched nutritional therapy in cancer patients with primary tumors at different sites. The results of this clinical study showed that hypercaloric, hyperproteic leucine-enriched oral supplementation did not provide significant improvement in the quality of life of cancer patients compared with a standard hypercaloric, whey protein-based hyperproteic oral supplementation [67]. However, studies raised the concerns that Leu supplementation also promotes cancer growth and aggressiveness [68,69,70], suggesting that Leu could have double-edged effects: anti-tumor and pro-tumorigenic (Table 1).

### 3.1. Anti-Tumor Effects of Leucine

Recent investigations have explored the use of Leu as a potential adjunct therapy in cancer treatment, focusing on its effects in tumor-bearing models and various cancer cell lines.

**Figure 2:**
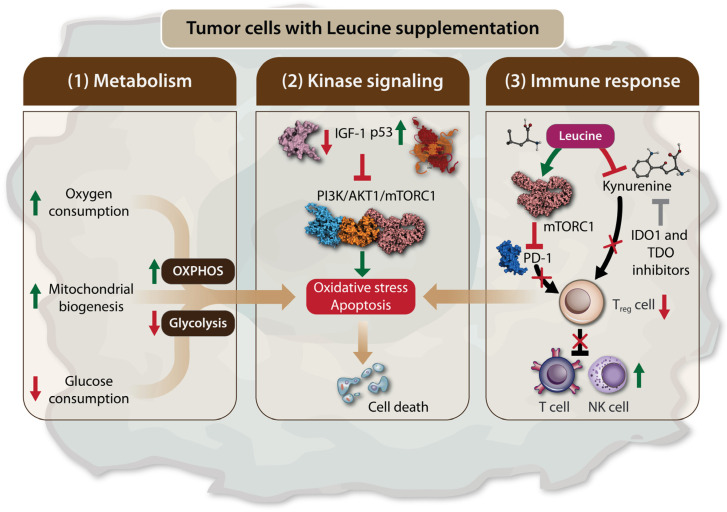
Leu supplementation leads to tumor cell death by inducing apoptosis and oxidative stress. Leu supplementation can lead to tumor cells death via the following three mechanisms: (1) The modulation of tumor cell metabolism. Leu supplementation favors oxidative phosphorylation (OXPHOS) over glycolysis by increasing oxygen consumption and mitochondrial biogenesis, while reducing glucose consumption. This metabolic shift promotes apoptosis and oxidative stress in tumor cells; (2) The regulation of the mTORC1 signaling through kinase pathway. Leu supplementation inhibits the expression of insulin-like growth factor 1 (IGF-1) and increases p53 levels. This results in the inhibition of the PI3K/AKT1/mTORC1 signaling axis in tumor cells, leading to apoptosis and tumor cell death; (3) The modulation of the immune response. Leu supplementation enhances anti-tumor immunity by inhibiting immunosuppressive regulatory T cells. This effect is mediated by mTORC1 activation, which leads to the inhibition of programmed cell death protein 1 (PD-1). Additionally, Leu competes with kynurenine in the kynurenine pathway (KP), thereby restoring the antitumor effects of T cells and natural killer (NK) cells. Therefore, the inhibitory effect of Leu, in combination with indoleamine 2,3-dioxygenase 1 (IDO1) and tryptophan 2,3-dioxygenase (TDO) inhibitors on kynurenine. Arrows in green stand for “activation”, “stimulation”, “increase in level”. Arrows in red stand for “decease in level”. Red bars and crosses stand for “inhibition”.

Viana et al. observed that a diet rich in Leu led to a metabolic shift of the Walker 256 rat tumor towards a less glycolytic profile [65]. This shift resulted in a reduced tumor glucose uptake and decreased tumor aggressiveness and metastatic sites in rats, but with no significant change in tumor size. Both Walker 256 rat tumor cells and tumor biopsies from rats fed the Leu-rich diet showed an increase in oxygen consumption, which was accompanied by an upregulation of mitochondrial genes, including *proliferator-activated receptor γ coactivator-1α* (*PGC-1α*), *nuclear respiratory factor-1* (*NRF-1*), *cyclooxygenase* (*COX*) *5a*, *citrate synthetase* (*CS*), and *cytochrome C*, indicating enhanced mitochondrial biogenesis and oxidative phosphorylation (OXPHOS) (Figure 2). These findings suggest that Leu induces a metabolic shift in Walker 256 tumors, favoring OXPHOS over glycolysis, both in vitro and in vivo [65]. The cytotoxic effects of L-Leu supplementation on cancer have been demonstrated in hepatocellular carcinoma (HCC) cell lines [72]. In these studies, Leu supplementation exerted a dose-dependent cytotoxic effect and induced apoptosis, which was attributed to a decrease in insulin-like growth factor 1 (IGF-1) levels and an increase in p53 levels, leading to the inhibition of the PI3K/AKT1/mTORC1 signaling pathway (Figure 2). The combination of Leu supplementation with indoleamine 2,3-dioxygenase 1 (IDO1) and tryptophan 2,3-dioxygenase (TDO) inhibitors has been proposed as a promising novel therapeutic approach. IDO1 and TDO are enzymes involved in the catabolism of tryptophan into kynurenine, a substrate in the kynurenine pathway (KP), which is known for its potent immunomodulatory effects that cancer cells exploit to evade immune system destruction (Figure 2) [79,80,81,82]. Leu, as a substrate of the system L transporter, competes with kynurenine. Consequently, Leu limits the powerful immunosuppressive effects of the KP and restores antitumor immunity when used in conjunction with IDO1 and TDO inhibitors [83]. Additionally, kynurenine interacts with transcription factors such as the aryl hydrocarbon receptor, which promotes the differentiation and activation of immunosuppressive T-regulatory cells, while inhibiting the proliferation of T cells and natural killer cells by increasing the expression of programmed cell death protein 1 (PD-1), a regulatory checkpoint molecule for T cells [84,85,86,87]. A recent study further supported the antitumor effects of Leu, particularly when combined with PD-1 inhibition. This combination was shown to reverse immune regulation and enhance the antitumor activity of CD8+ tumor-infiltrating lymphocytes (TILs), mediated by the activation of the mTORC1 signaling pathway (Figure 2) [75]. Another study demonstrated that a leucine-rich diet combined with anti-PD-1 therapy showed potent antitumor efficacy in lung, colorectal, and liver cancer models. This effect was associated with leucine’s ability to upregulate MHC-II genes in CD74+ neutrophils via the acetyl-CoA/H3K27ac/MHC-II axis, leading to enhanced antigen presentation and increased T cell infiltration into tumors [73]. The inhibitory effect of Leu, in combination with IDO1 and TDO inhibitors on kynurenine, represents a promising therapeutic approach for the future.

### 3.2. Pro-Tumorigenic Effects of Leucine

**Figure 3:**
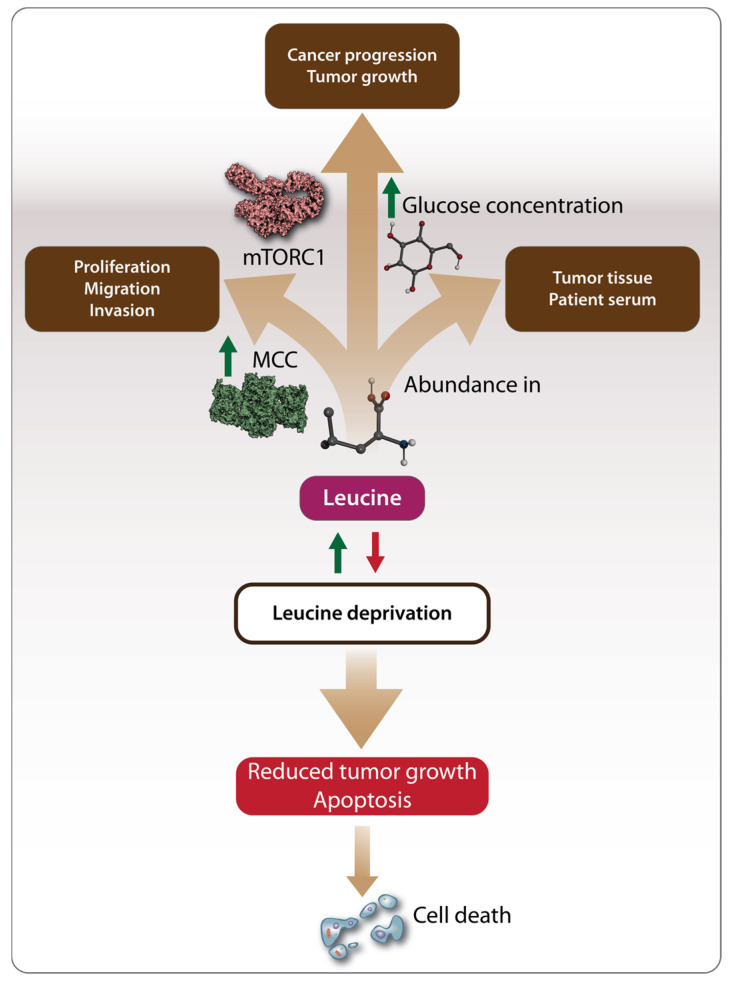
Mechanisms driving the pro-oncogenic effects of Leu. Methylcrotonoyl-CoA carboxylase (MCC) regulates Leu metabolism promoting tumor cell proliferation, migration, and invasion. Additionally, Leu provides energy to tumor cells by activating the mTORC1 pathway and facilitating glucose uptake, which serves as a necessary energy source for tumor cells. Leu has been found to be abundant in both tumor tissues and the serum of patients with hepatocellular carcinoma (HCC). Consequently, Leu deprivation has been proposed as a potential cancer treatment strategy leading to reduced tumor growth and apoptosis. Arrows in green stand for “activation”, “stimulation”, “increase in level”, “leucine supplementation”. Arrows in red stand for “leucine deprivation”.

Although the studies mentioned above in Section 3.1 highlighted the anticancer effects of Leu, an increasing body of research suggests that Leu may also have pro-oncogenic effects. Like other amino acids, Leu can serve as an energy source, potentially fueling cancer development [88]. The pro-oncogenic effects of Leu have been studied in various cancers, including hepatocellular carcinoma (HCC), breast cancer, and pancreatic cancer (Table 1). In contrast to the findings of Hassan et al. [72], Chen et al. [74] demonstrated that the growth of HCC cell lines depends on the presence of Leu, with deprivation significantly reducing the proliferation, migration, and invasion of cancer cells. This pro-oncogenic effect was linked to the regulation of Leu metabolism by enzymes such as the mitochondrial methylcrotonoyl-CoA carboxylase (MCC) enzyme, which is highly expressed and associated with poor prognosis in HCC patients [74] (Figure 3). The pro-oncogenic role of Leu in HCC has also been supported by recent metabolomics studies, which identified Leu as one of the amino acids significantly upregulated in both HCC tumor tissues and the serum of HCC patients [89]. Another recent study using mendelian randomization (MR) methods explored the possible causal relationship between genetically predicted circulating BCAA concentrations and various cancers by leveraging the large datasets for BCAAs provided by genome-wide association studies (GWAS). The results showed elevated circulating total BCAA levels and leucine levels, indicating a positive association between leucine and squamous cell lung cancer [12].

As a result, dietary amino acid supplementation has become an increasingly researched area as a potential cancer treatment strategy (Figure 3). In vitro studies on breast cancer have shown that Leu deprivation inhibits cancer cell proliferation and induces apoptosis, while in vivo studies demonstrated inhibited tumor growth [71]. Another recent study on breast cancer found that a high-fat diet, leading to the abundant release of Leu, promoted cancer progression in tumor-bearing mice. This effect was mediated by the activation of the mTOR signaling pathway, which led to the differentiation and infiltration of polymorphonuclear myeloid-derived suppressor cells, a factor associated with poor clinical outcomes in breast cancer patients [77]. Complementing these observations, the pro-oncogenic effects of Leu have also been demonstrated in bladder cancer, pancreatic, and colorectal cancer. Long-term supplementation with excessive amounts of Leu promoted bladder carcinogenesis in rats [70,78], suggesting that both the duration and amount of supplementation play a role in Leu’s oncogenic potential. In pancreatic cancer, Leu supplementation increased tumor growth in both lean and overweight mice, but through different mechanisms. In lean mice, Leu promoted tumor growth by activating the mTOR signaling pathway, while, in overweight mice, Leu supplementation increased the amount of glucose available to tumor cells, further accelerating tumor growth (Figure 3) [68].

In a separate study, Leu was found to promote the growth and proliferation of pancreatic cancer cells by stimulating the expression of SESN2 and increasing phosphorylated mTOR (p-mTOR), indicating the involvement of the mTOR signaling pathway in pancreatic cancer development. The use of mTOR inhibitors and SESN2 expression vectors in this study clearly demonstrated that SESN2 promotes glycolysis in pancreatic cancer through the mTOR signaling pathway (Figure 3) [73].

In colorectal cancer, leucine plays a pro-tumorigenic role by stimulating a subset of regulatory B cells that express leucine–tRNA synthetase-2 (LARS2) [76]. These cells are associated with colorectal hyperplasia and decreased survival in colorectal cancer patients. The underlying mechanism involves cancer immune evasion via transforming growth factor beta-1 (TGF-β1) secretion by LARS B cells, driven by leucine-stimulated mitochondrial NAD+ regeneration and oxidative metabolism [76]. As a result, the study proposed a leucine-restricted diet as a therapeutic approach for colorectal cancer, which was shown to suppress cancer immune evasion [76].

## 4. Conclusions

Leu, as a BCAA, has become one of the most popular sport supplements due to its role in promoting MPS [27]. In the light of this, Leu and its metabolites have been found to improve muscle wasting in CC [90]. However, the role of Leu remains controversial in certain cancers [72,74]. Through this review of the literature, Leu has been identified as a ‘dichotomous’ amino acid, exhibiting both anticancer and pro-tumorigenic effects. In conclusion, Leu has been found to exhibit significant anticancer activity [65,72,75,83], leading to cell death by regulating tumor cell metabolism, apoptosis, and immune signaling pathways. On the other hand, Leu also demonstrates pro-oncogenic activity, promoting tumor cell proliferation through various mechanisms depending on the cancer type. For example, Leu can regulate its own metabolism in HCC [74,89], increase glucose availability to tumor cells, or activate the mTOR signaling pathway in pancreatic, breast, and bladder cancers [68,71,77,78,91]. The pro-tumorigenic effects of Leu may be achieved in several ways such as by supporting the metabolic reprogramming of cancer cells, similar to other amino acids [92], or by stimulating tumorigenesis through the modulation of oncogenic signaling pathways [93]. Additionally, the duration and amount of Leu supplementation appear to influence its pro-tumorigenic activity. It was investigated in animal experiments and demonstrated that a high dose of leucine supplementation for long-term promotes carcinogenesis [70,78]. Concerns about the adverse effects of leucine with excess amount intake have been raised. Just recently, tolerable upper intake levels (ULs) of individual amino acids were recapitulated using well-conducted studies of human-dose response trials. The ULs for leucine were identified to be 35 g/day for the young and 30 g/day for the elderly, respectively [94]. More preclinical and clinical trials are needed to investigate the effects of leucine, either anti-tumor or pro-tumorigenic, along with focusing on the intake amount and duration. The dual nature of Leu in cancer biology reveals its complexity and emphasizes the need for careful evaluation. The future use of Leu, either as a nutritional supplement or a potential cancer treatment, will depend on further research clarifying its effects. Moreover, suggestions on targeting enzymes involved in Leu metabolism and Leu dietary interventions as treatment strategies may present challenges, which may arise due to the complex nature of Leu metabolism, individual variability in response, potential side effects, and limited research on long-term effects and optimal dosages.

## Figures and Tables

**Table 1:** Summary information on the antitumor or pro-tumorigenic action of leucine intervention in various types of cancer.

Effects of Leucine	Cancer Type	Leucine Intervention	Mechanisms of Action	Source(s)
Antitumor effects	Breast cancer	Leu deprivation	Increased caspase activity	[71]
	Liver cancer	L-Leu supplementation	Induced apoptosis	[72]
		Leu supplementation	Increased histone acetylation and expression of antigen-presenting genes in CD74+ neutrophils	[73]
		Leu deprivation	Decreased leucine metabolism	[74]
	Colon cancer	Leu supplementation	Activated mTORC1 in CD8+ TILs	[75]
	Colorectal cancer	Leu supplementation	Increased histone acetylation and expression of antigen-presenting genes in CD74+ neutrophils	[73]
		Leu deprivation	Limited TGF-β1 secretion in LARS B cells	[76]
	Lung cancer	Leu supplementation	Increased histone acetylation and expression of antigen-presenting genes in CD74+ neutrophils	[73]
Pro-tumorigenic effects	Breast cancer	High-fat diet (Leu releasing)	Activated mTOR signaling	[77]
	Bladder cancer	Leu supplementation	Activated amino-acid transporters and oncogenic genes	[70,78]
	Pancreatic cancer	Leu supplementation	Activated mTOR signaling or elevated glucose levels in the bloodstream	[68,71]
	Colorectal cancer	Leu supplementation	Increased LARS B cell generationIncreased TGF-β1 production	[75]

## Data Availability

All relevant data are available within the article.

## References

[1] Walsh C.T., O’Brien R.V., Khosla C. (2013). Nonproteinogenic Amino Acid Building Blocks for Nonribosomal Peptide and Hybrid Polyketide Scaffolds. Angew. Chem. Int. Ed..

[2] Thalacker-Mercer A., Riddle E., Barre L. (2020). Protein and Amino Acids for Skeletal Muscle Health in Aging. Advances in Food and Nutrition Research.

[3] Durante W., Wu G. (2020). Amino Acids in Circulatory Function and Health. Amino Acids in Nutrition and Health.

[4] Beaumont M., Blachier F., Wu G. (2020). Amino Acids in Intestinal Physiology and Health. Amino Acids in Nutrition and Health.

[5] Flynn N.E., Shaw M.H., Becker J.T., Wu G. (2020). Amino Acids in Health and Endocrine Function. Amino Acids in Nutrition and Health.

[6] Li P., Yin Y.-L., Li D., Woo Kim S., Wu G. (2007). Amino Acids and Immune Function. Br. J. Nutr..

[7] Dimou A., Tsimihodimos V., Bairaktari E. (2022). The Critical Role of the Branched Chain Amino Acids (BCAAs) Catabolism-Regulating Enzymes, Branched-Chain Aminotransferase (BCAT) and Branched-Chain α-Keto Acid Dehydrogenase (BCKD), in Human Pathophysiology. Int. J. Mol. Sci..

[8] Neinast M., Murashige D., Arany Z. (2019). Branched Chain Amino Acids. Annu. Rev. Physiol..

[9] Zhang S., Zeng X., Ren M., Mao X., Qiao S. (2017). Novel Metabolic and Physiological Functions of Branched Chain Amino Acids: A Review. J. Anim. Sci. Biotechnol..

[10] Holeček M. (2018). Branched-Chain Amino Acids in Health and Disease: Metabolism, Alterations in Blood Plasma, and as Supplements. Nutr. Metab..

[11] Jung M.K., Okekunle A.P., Lee J.E., Sung M.K., Lim Y.J. (2021). Role of Branched-Chain Amino Acid Metabolism in Tumor Development and Progression. J. Cancer Prev..

[12] Xu H., Wang X., Xu X., Liu L., Zhang Y., Yan X., Zhang Y., Dang K., Li Y. (2023). Association of Plasma Branched-Chain Amino Acid with Multiple Cancers: A Mendelian Randomization Analysis. Clin. Nutr..

[13] Lal H., Chugh K. (1995). Metabolic and Regulatory Effects of Branched Chain Amino Acid Supplementation. Nutr. Res..

[14] May M.E., Buse M.G. (1989). Effects of Branched-Chain Amino Acids on Protein Turnover. Diabetes Metab. Rev..

[15] Nelson A.R., Phillips S.M., Stellingwerff T., Rezzi S., Bruce S.J., Breton I., Thorimbert A., Guy P.A., Clarke J., Broadbent S. (2012). A Protein–Leucine Supplement Increases Branched-Chain Amino Acid and Nitrogen Turnover but Not Performance. Med. Sci. Sports Exerc..

[16] Churchward-Venne T.A., Burd N.A., Phillips S.M. (2012). Nutritional Regulation of Muscle Protein Synthesis with Resistance Exercise: Strategies to Enhance Anabolism. Nutr. Metab..

[17] Jackman S.R., Witard O.C., Philp A., Wallis G.A., Baar K., Tipton K.D. (2017). Branched-Chain Amino Acid Ingestion Stimulates Muscle Myofibrillar Protein Synthesis Following Resistance Exercise in Humans. Front. Physiol..

[18] Wang C., Guo F. (2013). Branched Chain Amino Acids and Metabolic Regulation. Chin. Sci. Bull..

[19] Lynch C.J., Adams S.H. (2014). Branched-Chain Amino Acids in Metabolic Signalling and Insulin Resistance. Nat. Rev. Endocrinol..

[20] Rahimi M.H., Shab-Bidar S., Mollahosseini M., Djafarian K. (2017). Branched-Chain Amino Acid Supplementation and Exercise-Induced Muscle Damage in Exercise Recovery: A Meta-Analysis of Randomized Clinical Trials. Nutrition.

[21] Blomstrand E. (2006). A Role for Branched-Chain Amino Acids in Reducing Central Fatigue. J. Nutr..

[22] Gervasi M., Sisti D., Amatori S., Donati Zeppa S., Annibalini G., Piccoli G., Vallorani L., Benelli P., Rocchi M.B.L., Barbieri E. (2020). Effects of a Commercially Available Branched-Chain Amino Acid-Alanine-Carbohydrate-Based Sports Supplement on Perceived Exertion and Performance in High Intensity Endurance Cycling Tests. J. Int. Soc. Sports Nutr..

[23] Wolfe R.R. (2017). Branched-Chain Amino Acids and Muscle Protein Synthesis in Humans: Myth or Reality?. J. Int. Soc. Sports Nutr..

[24] Global BCAA Market [2024–2032] | Advanced Research Report. https://www.linkedin.com/pulse/global-bcaa-market-2024-2032-advanced-research-ipnaf/.

[25] Gleeson M. (2005). Interrelationship between Physical Activity and Branched-Chain Amino Acids. J. Nutr..

[26] Marcon M., Zanella P.B. (2022). The Effect of Branched-Chain Amino Acids Supplementation in Physical Exercise: A Systematic Review of Human Randomized Controlled Trials. Sci. Sports.

[27] Plotkin D.L., Delcastillo K., Van Every D.W., Tipton K.D., Aragon A.A., Schoenfeld B.J. (2021). Isolated Leucine and Branched-Chain Amino Acid Supplementation for Enhancing Muscular Strength and Hypertrophy: A Narrative Review. Int. J. Sport Nutr. Exerc. Metab..

[28] Han L., Dong L., Leung K., Zhao Z., Li Y., Gao L., Chen Z., Xue J., Qing Y., Li W. (2023). METTL16 Drives Leukemogenesis and Leukemia Stem Cell Self-Renewal by Reprogramming BCAA Metabolism. Cell Stem Cell.

[29] Lee J.H., Cho Y., Kim J.H., Kim J., Nam H.Y., Kim S.W., Son J. (2019). Branched-Chain Amino Acids Sustain Pancreatic Cancer Growth by Regulating Lipid Metabolism. Exp. Mol. Med..

[30] Columbus D.A., Fiorotto M.L., Davis T.A. (2015). Leucine Is a Major Regulator of Muscle Protein Synthesis in Neonates. Amino Acids.

[31] Rehman S.U., Ali R., Zhang H., Zafar M.H., Wang M. (2023). Research Progress in the Role and Mechanism of Leucine in Regulating Animal Growth and Development. Front. Physiol..

[32] Kang M.C. (2020). Muscle Protein Metabolism in Critically Illness. Surg. Metab. Nutr..

[33] Zaromskyte G., Prokopidis K., Ioannidis T., Tipton K.D., Witard O.C. (2021). Evaluating the Leucine Trigger Hypothesis to Explain the Post-Prandial Regulation of Muscle Protein Synthesis in Young and Older Adults: A Systematic Review. Front. Nutr..

[34] Wilkinson D.J., Hossain T., Hill D.S., Phillips B.E., Crossland H., Williams J., Loughna P., Churchward-Venne T.A., Breen L., Phillips S.M. (2013). Effects of Leucine and Its Metabolite Β-hydroxy-β-methylbutyrate on Human Skeletal Muscle Protein Metabolism. J. Physiol..

[35] Ananieva E.A., Powell J.D., Hutson S.M. (2016). Leucine Metabolism in T Cell Activation: mTOR Signaling and Beyond. Adv. Nutr..

[36] Duan Y., Li F., Song B., Zheng C., Zhong Y., Xu K., Kong X., Yin Y., Wang W., Shu G. (2019). β-Hydroxy-β-Methyl Butyrate, but Not α-Ketoisocaproate and Excess Leucine, Stimulates Skeletal Muscle Protein Metabolism in Growing Pigs Fed Low-Protein Diets. J. Funct. Foods.

[37] Escobar J., Frank J.W., Suryawan A., Nguyen H.V., Van Horn C.G., Hutson S.M., Davis T.A. (2010). Leucine and α-Ketoisocaproic Acid, but Not Norleucine, Stimulate Skeletal Muscle Protein Synthesis in Neonatal Pigs. J. Nutr..

[38] Cereda E., Pisati R., Rondanelli M., Caccialanza R. (2022). Whey Protein, Leucine- and Vitamin-D-Enriched Oral Nutritional Supplementation for the Treatment of Sarcopenia. Nutrients.

[39] Gielen E., Beckwée D., Delaere A., De Breucker S., Vandewoude M., Bautmans I., Bautmans I., Beaudart C., Beckwée D., the Sarcopenia Guidelines Development Group of the Belgian Society of Gerontology and Geriatrics (BSGG) (2021). Nutritional Interventions to Improve Muscle Mass, Muscle Strength, and Physical Performance in Older People: An Umbrella Review of Systematic Reviews and Meta-Analyses. Nutr. Rev..

[40] Martínez-Arnau F.M., Fonfría-Vivas R., Buigues C., Castillo Y., Molina P., Hoogland A.J., van Doesburg F., Pruimboom L., Fernández-Garrido J., Cauli O. (2020). Effects of Leucine Administration in Sarcopenia: A Randomized and Placebo-Controlled Clinical Trial. Nutrients.

[41] Yoshimura Y., Bise T., Shimazu S., Tanoue M., Tomioka Y., Araki M., Nishino T., Kuzuhara A., Takatsuki F. (2019). Effects of a Leucine-Enriched Amino Acid Supplement on Muscle Mass, Muscle Strength, and Physical Function in Post-Stroke Patients with Sarcopenia: A Randomized Controlled Trial. Nutrition.

[42] Bodine S.C. (2022). The Role of mTORC1 in the Regulation of Skeletal Muscle Mass. Fac. Rev..

[43] Suryawan A., Rudar M., Fiorotto M.L., Davis T.A. (2020). Differential Regulation of mTORC1 Activation by Leucine and β-Hydroxy-β-Methylbutyrate in Skeletal Muscle of Neonatal Pigs. J. Appl. Physiol..

[44] Akbay B., Shmakova A., Vassetzky Y., Dokudovskaya S. (2020). Modulation of mTORC1 Signaling Pathway by HIV-1. Cells.

[45] Nicklin P., Bergman P., Zhang B., Triantafellow E., Wang H., Nyfeler B., Yang H., Hild M., Kung C., Wilson C. (2009). Bidirectional Transport of Amino Acids Regulates mTOR and Autophagy. Cell.

[46] Chantranupong L., Wolfson R.L., Orozco J.M., Saxton R.A., Scaria S.M., Bar-Peled L., Spooner E., Isasa M., Gygi S.P., Sabatini D.M. (2014). The Sestrins Interact with GATOR2 to Negatively Regulate the Amino-Acid-Sensing Pathway Upstream of mTORC1. Cell Rep..

[47] Han J.M., Jeong S.J., Park M.C., Kim G., Kwon N.H., Kim H.K., Ha S.H., Ryu S.H., Kim S. (2012). Leucyl-tRNA Synthetase Is an Intracellular Leucine Sensor for the mTORC1-Signaling Pathway. Cell.

[48] Kim J.S., Ro S.-H., Kim M., Park H.-W., Semple I.A., Park H., Cho U.-S., Wang W., Guan K.-L., Karin M. (2015). Sestrin2 Inhibits mTORC1 through Modulation of GATOR Complexes. Sci. Rep..

[49] Parmigiani A., Nourbakhsh A., Ding B., Wang W., Kim Y.C., Akopiants K., Guan K.-L., Karin M., Budanov A.V. (2014). Sestrins Inhibit mTORC1 Kinase Activation through the GATOR Complex. Cell Rep..

[50] Saxton R.A., Knockenhauer K.E., Wolfson R.L., Chantranupong L., Pacold M.E., Wang T., Schwartz T.U., Sabatini D.M. (2016). Structural Basis for Leucine Sensing by the Sestrin2-mTORC1 Pathway. Science.

[51] Wolfson R.L., Chantranupong L., Saxton R.A., Shen K., Scaria S.M., Cantor J.R., Sabatini D.M. (2016). Sestrin2 Is a Leucine Sensor for the mTORC1 Pathway. Science.

[52] Yang M., Lu Y., Piao W., Jin H. (2022). The Translational Regulation in mTOR Pathway. Biomolecules.

[53] Moghei M., Tavajohi-Fini P., Beatty B., Adegoke O.A.J. (2016). Ketoisocaproic Acid, a Metabolite of Leucine, Suppresses Insulin-Stimulated Glucose Transport in Skeletal Muscle Cells in a BCAT2-Dependent Manner. Am. J. Physiol.-Cell Physiol..

[54] Kao M., Columbus D.A., Suryawan A., Steinhoff-Wagner J., Hernandez-Garcia A., Nguyen H.V., Fiorotto M.L., Davis T.A. (2016). Enteral β-Hydroxy-β-Methylbutyrate Supplementation Increases Protein Synthesis in Skeletal Muscle of Neonatal Pigs. Am. J. Physiol.-Endocrinol. Metab..

[55] Wilkinson D.J., Hossain T., Limb M.C., Phillips B.E., Lund J., Williams J.P., Brook M.S., Cegielski J., Philp A., Ashcroft S. (2018). Impact of the Calcium Form of β-Hydroxy-β-Methylbutyrate upon Human Skeletal Muscle Protein Metabolism. Clin. Nutr..

[56] Girón M.D., Vílchez J.D., Salto R., Manzano M., Sevillano N., Campos N., Argilés J.M., Rueda R., López-Pedrosa J.M. (2016). Conversion of Leucine to β-Hydroxy-β-Methylbutyrate by α-Keto Isocaproate Dioxygenase Is Required for a Potent Stimulation of Protein Synthesis in L6 Rat Myotubes: HMB Is a Potent Stimulator of Protein Synthesis. J. Cachexia Sarcopenia Muscle.

[57] Huang H., Long L., Zhou P., Chapman N.M., Chi H. (2020). mTOR Signaling at the Crossroads of Environmental Signals and T-cell Fate Decisions. Immunol. Rev..

[58] Manning B.D., Toker A. (2017). AKT/PKB Signaling: Navigating the Network. Cell.

[59] Cai S.-L., Tee A.R., Short J.D., Bergeron J.M., Kim J., Shen J., Guo R., Johnson C.L., Kiguchi K., Walker C.L. (2006). Activity of TSC2 Is Inhibited by AKT-Mediated Phosphorylation and Membrane Partitioning. J. Cell Biol..

[60] Dibble C.C., Elis W., Menon S., Qin W., Klekota J., Asara J.M., Finan P.M., Kwiatkowski D.J., Murphy L.O., Manning B.D. (2012). TBC1D7 Is a Third Subunit of the TSC1-TSC2 Complex Upstream of mTORC1. Mol. Cell.

[61] Garlick P.J. (2005). The Role of Leucine in the Regulation of Protein Metabolism. J. Nutr..

[62] Ham D.J., Caldow M.K., Lynch G.S., Koopman R. (2014). Leucine as a Treatment for Muscle Wasting: A Critical Review. Clin. Nutr..

[63] Beaudry A.G., Law M.L. (2022). Leucine Supplementation in Cancer Cachexia: Mechanisms and a Review of the Pre-Clinical Literature. Nutrients.

[64] Cruz B., Oliveira A., Gomes-Marcondes M.C.C. (2017). L-Leucine Dietary Supplementation Modulates Muscle Protein Degradation and Increases pro-Inflammatory Cytokines in Tumour-Bearing Rats. Cytokine.

[65] Viana L.R., Tobar N., Busanello E.N.B., Marques A.C., De Oliveira A.G., Lima T.I., Machado G., Castelucci B.G., Ramos C.D., Brunetto S.Q. (2019). Leucine-Rich Diet Induces a Shift in Tumour Metabolism from Glycolytic towards Oxidative Phosphorylation, Reducing Glucose Consumption and Metastasis in Walker-256 Tumour-Bearing Rats. Sci. Rep..

[66] Maschke J., Kruk U., Kastrati K., Kleeberg J., Buchholz D., Erickson N., Huebner J. (2017). Nutritional Care of Cancer Patients: A Survey on Patients’ Needs and Medical Care in Reality. Int. J. Clin. Oncol..

[67] Herrera-Martínez A.D., León Idougourram S., Muñoz Jiménez C., Rodríguez-Alonso R., Alonso Echague R., Chica Palomino S., Sanz Sanz A., Manzano García G., Gálvez Moreno M.Á., Calañas Continente A. (2023). Standard Hypercaloric, Hyperproteic vs. Leucine-Enriched Oral Supplements in Patients with Cancer-Induced Sarcopenia, a Randomized Clinical Trial. Nutrients.

[68] Liu K.A., Lashinger L.M., Rasmussen A.J., Hursting S.D. (2014). Leucine Supplementation Differentially Enhances Pancreatic Cancer Growth in Lean and Overweight Mice. Cancer Metab..

[69] Schrems E.R., Haynie W.S., Perry R.A., Morena F., Cabrera A.R., Rosa-Caldwell M.E., Greene N.P., Washington T.A. (2023). Leucine Supplementation Exacerbates Morbidity in Male but Not Female Mice with Colorectal Cancer-Induced Cachexia. Nutrients.

[70] Xie X.-L., Wei M., Yunoki T., Kakehashi A., Yamano S., Kato M., Wanibuchi H. (2012). Long-Term Treatment with l-Isoleucine or l-Leucine in AIN-93G Diet Has Promoting Effects on Rat Bladder Carcinogenesis. Food Chem. Toxicol..

[71] Xiao F., Wang C., Yin H., Yu J., Chen S., Fang J., Guo F. (2016). Leucine Deprivation Inhibits Proliferation and Induces Apoptosis of Human Breast Cancer Cells via Fatty Acid Synthase. Oncotarget.

[72] Hassan Y.A., Helmy M.W., Ghoneim A.I. (2021). Combinatorial Antitumor Effects of Amino Acids and Epigenetic Modulations in Hepatocellular Carcinoma Cell Lines. Naunyn-Schmiedeberg’s Arch. Pharmacol..

[73] Wu Y., Ma J., Yang X., Nan F., Zhang T., Ji S., Rao D., Feng H., Gao K., Gu X. (2024). Neutrophil Profiling Illuminates Anti-Tumor Antigen-Presenting Potency. Cell.

[74] Chen Y.-Y., Zhang X.-N., Xu C.-Z., Zhou D.-H., Chen J., Liu Z.-X., Sun Y., Huang W., Qu L.-S. (2021). MCCC2 Promotes HCC Development by Supporting Leucine Oncogenic Function. Cancer Cell Int..

[75] Zhang Y., Hu H., Liu W., Yan S.-M., Li Y., Tan L., Chen Y., Liu J., Peng Z., Yuan Y. (2021). Amino Acids and RagD Potentiate mTORC1 Activation in CD8 ^+^ T Cells to Confer Antitumor Immunity. J. Immunother. Cancer.

[76] Wang Z., Lu Z., Lin S., Xia J., Zhong Z., Xie Z., Xing Y., Qie J., Jiao M., Li Y. (2022). Leucine-tRNA-Synthetase-2-Expressing B Cells Contribute to Colorectal Cancer Immunoevasion. Immunity.

[77] Chen J., Liu X., Zou Y., Gong J., Ge Z., Lin X., Zhang W., Huang H., Zhao J., Saw P.E. (2024). A High-Fat Diet Promotes Cancer Progression by Inducing Gut Microbiota–Mediated Leucine Production and PMN-MDSC Differentiation. Proc. Natl. Acad. Sci. USA.

[78] Gi M., Wanibuchi H. (2015). Roles of Leucine and Isoleucine in Experimental Models of Bladder Carcinogenesis. Food Saf..

[79] Ball H.J., Fedelis F.F., Bakmiwewa S.M., Hunt N.H., Yuasa H.J. (2014). Tryptophan-Catabolizing Enzymes—Party of Three. Front. Immunol..

[80] Hoffmann D., Dvorakova T., Stroobant V., Bouzin C., Daumerie A., Solvay M., Klaessens S., Letellier M.-C., Renauld J.-C., Van Baren N. (2020). Tryptophan 2,3-Dioxygenase Expression Identified in Human Hepatocellular Carcinoma Cells and in Intratumoral Pericytes of Most Cancers. Cancer Immunol. Res..

[81] Pilotte L., Larrieu P., Stroobant V., Colau D., Dolušić E., Frédérick R., De Plaen E., Uyttenhove C., Wouters J., Masereel B. (2012). Reversal of Tumoral Immune Resistance by Inhibition of Tryptophan 2,3-Dioxygenase. Proc. Natl. Acad. Sci. USA.

[82] Uyttenhove C., Pilotte L., Théate I., Stroobant V., Colau D., Parmentier N., Boon T., Van Den Eynde B.J. (2003). Evidence for a Tumoral Immune Resistance Mechanism Based on Tryptophan Degradation by Indoleamine 2,3-Dioxygenase. Nat. Med..

[83] Kim M., Tomek P. (2021). Tryptophan: A Rheostat of Cancer Immune Escape Mediated by Immunosuppressive Enzymes IDO1 and TDO. Front. Immunol..

[84] Chiesa M.D., Carlomagno S., Frumento G., Balsamo M., Cantoni C., Conte R., Moretta L., Moretta A., Vitale M. (2006). The Tryptophan Catabolite L-Kynurenine Inhibits the Surface Expression of NKp46- and NKG2D-Activating Receptors and Regulates NK-Cell Function. Blood.

[85] Liu Y., Liang X., Dong W., Fang Y., Lv J., Zhang T., Fiskesund R., Xie J., Liu J., Yin X. (2018). Tumor-Repopulating Cells Induce PD-1 Expression in CD8+ T Cells by Transferring Kynurenine and AhR Activation. Cancer Cell.

[86] Mezrich J.D., Fechner J.H., Zhang X., Johnson B.P., Burlingham W.J., Bradfield C.A. (2010). An Interaction between Kynurenine and the Aryl Hydrocarbon Receptor Can Generate Regulatory T Cells. J. Immunol..

[87] Rad Pour S., Morikawa H., Kiani N.A., Yang M., Azimi A., Shafi G., Shang M., Baumgartner R., Ketelhuth D.F.J., Kamleh M.A. (2019). Exhaustion of CD4+ T-Cells Mediated by the Kynurenine Pathway in Melanoma. Sci. Rep..

[88] Keenan M.M., Chi J.-T. (2015). Alternative Fuels for Cancer Cells. Cancer J..

[89] Morine Y., Utsunomiya T., Yamanaka-Okumura H., Saito Y., Yamada S., Ikemoto T., Imura S., Kinoshita S., Hirayama A., Tanaka Y. (2022). Essential Amino Acids as Diagnostic Biomarkers of Hepatocellular Carcinoma Based on Metabolic Analysis. Oncotarget.

[90] Aversa Z., Costelli P., Muscaritoli M. (2017). Cancer-Induced Muscle Wasting: Latest Findings in Prevention and Treatment. Ther. Adv. Med. Oncol..

[91] Guo Y., Zhu H., Weng M., Zhang H., Wang C., Sun L. (2020). CC-223, NSC781406, and BGT226 Exerts a Cytotoxic Effect Against Pancreatic Cancer Cells via mTOR Signaling. Front. Pharmacol..

[92] Wei Z., Liu X., Cheng C., Yu W., Yi P. (2021). Metabolism of Amino Acids in Cancer. Front. Cell Dev. Biol..

[93] Chen J., Cui L., Lu S., Xu S. (2024). Amino Acid Metabolism in Tumor Biology and Therapy. Cell Death Dis..

[94] Elango R. (2023). Tolerable Upper Intake Level for Individual Amino Acids in Humans: A Narrative Review of Recent Clinical Studies. Adv. Nutr..

